# Descriptive peer drinking norms and binge drinking: Enhancement motives as a mediator and alcohol resistance self-efficacy as a moderator

**DOI:** 10.3389/fpsyg.2022.876274

**Published:** 2022-10-11

**Authors:** Jianyong Chen, Yuzhi Li, Yaping Zhang, Ju Feng, Liang Jia

**Affiliations:** ^1^Department of Psychology, Zhejiang Normal University, Jinhua, Zhejiang, China; ^2^Key Laboratory of Intelligent Education Technology and Application of Zhejiang Province, Zhejiang Normal University, Jinhua, Zhejiang, China

**Keywords:** descriptive peer drinking norms, binge drinking, enhancement motives, alcohol resistance self-efficacy, lowerclassmen

## Abstract

The association between descriptive norms regarding peer drinking and college students’ binge drinking has been established; however, the mediating and moderating mechanisms underlying this relationship among first-and second-year college students remain minimally explored. Drawing on social norms theory, motivational model of alcohol use, and the theory of normative social behavior, the current study investigated whether enhancement drinking motives mediated the relationship between descriptive norms regarding peer drinking and college students’ binge drinking, and whether this relationship was moderated by alcohol resistance self-efficacy. Five hundred and nineteen first-and second-year college students (*M*_age_ = 19.19 years, *SD* = 0.98) who were from four universities and had at least one time of heavy episodic drinking during the last year completed self-report questionnaires. After controlling for sex, age, and university variable, stronger descriptive norms regarding peer drinking were positively associated with a greater frequency of binge drinking. Enhancement drinking motives partially mediated the effects of descriptive peer drinking norms on binge drinking. Furthermore, alcohol resistance self-efficacy moderated the direct effects of descriptive peer drinking norms on binge drinking. Compared with college students who reported high alcohol resistance self-efficacy, the direct effects of descriptive peer drinking norms on binge drinking were stronger among students with low alcohol resistance self-efficacy. These findings point to the potential value of alcohol intervention approaches including efforts to help first-and second-year college students change enhancement drinking motives and increase their ability of resisting drinks in the context of pervasive peer drinking.

## Introduction

Binge drinking among college students is a major public health concern, which can produce a variety of problems, including decreased academic achievement (Thombs et al., 2009), increased risk of assault or sexual assault and alcohol-related traffic accidents (Hingson et al., 2009), and deaths due to alcohol overdose (Hingson et al., 2017). The binge drinking is often defined as consuming 4 or more alcoholic drinks for females, and 5 or more drinks for males on one single occasion (National Institute on Alcohol Abuse and Alcoholism, 2015). In 2018, approximately 42% of Chinese full-time college students reported binge drinking during the past year (Sun et al., 2019; Liu et al., 2021). In addition, first-and second-year college students are at the transition from high school to college, and the first two years of college are considered a period when alcohol drinking is becoming established (e.g., a sizable proportion of college students experience changes from abstinence to drinking) and escalating (e.g., drinking is moving towards its peak; Lindgren et al., 2018). Thus, it is critical to investigate risk factors that could be incorporated into intervention efforts for binge drinking among college student drinkers during the critical transition into college.

### Descriptive norms regarding peer drinking and alcohol consumption

Individuals’ perceived drinking norms (i.e., the perceived beliefs regarding the prevalence and acceptability of drinking by others) may play a significant role in contributing to their alcohol use. The perceived drinking norms are often conceptually distinguished between descriptive norms and injunctive norms (Borsari and Carey, 2003; Lac and Donaldson, 2018). Descriptive norms refer to perceptions of frequency, quantity, and popularity of drinking by others, and are mainly based on observations of others’ alcohol consumption. Injunctive norms refer to perceptions about the extent that others approve of drinking, and assist an individual in determining whether alcohol drinking is acceptable or not. According to social norms theory (Berkowitz, 2005), the perceptions of behaviors and attitudes of reference groups influence individuals’ own behaviors. Consistent with this theory, previous findings have consistently demonstrated that college students who had a higher level of descriptive norms for alcohol use among others (e.g., friends, parents, and typical college students) were more likely to engage in alcohol drinking (McAlaney and McMahon, 2007; Neighbors et al., 2008; DiBello et al., 2018; Lac and Donaldson, 2018) and heavy drinking (Park et al., 2009; Franca et al., 2010) than those who perceived others to be abstinent. Additionally, the effects of *descriptive norms* on drinking behaviors vary by proximity of reference groups. The descriptive norms of proximal referents (e.g., friends and parents) exert a greater influence on college students’ alcohol use as compared to more distal referents (e.g., typical college students; Hagler et al., 2017; Lac and Donaldson, 2018). Moreover, correcting the misperceptions of drinking behaviors of peers or typical college students (i.e., descriptive norms) leads to reductions of drinking quantities and binge drinking episodes among college students (Dotson et al., 2015; Boyle et al., 2017). However, the relationship between *injunctive norms* and alcohol use is less consistent in college students. For example, some studies showed that injunctive drinking norms were associated with greater alcohol use regardless of the specific referents used (LaBrie et al., 2010; Collins and Spelman, 2013), whereas others revealed that injunctive norms for proximal and distal referents had opposite effects on alcohol consumption (Neighbors et al., 2008; Krieger et al., 2016; Lac and Donaldson, 2018). In light of the above findings and the fact that peer groups become more important when college students gain autonomy from parents, the present study focused on descriptive norms regarding peer drinking among Chinese college students.

Most prior research on the relation between descriptive norms regarding peer drinking and alcohol consumption has been conducted in Western countries, and it is uncertain whether the findings of such studies could be generalized to other cultures. In China, frequent toasting during meals with friends is very common, and the cultural norms emphasize the value of mutual intoxication through heavy alcohol use among adults, including college students (Cheng et al., 2017; Sun et al., 2018). Thus, heavy episodic drinking may become a pervasive and salient norm among Chinese college students, which may differ from that of Western countries. One aim of this study was to examine whether descriptive norms regarding peer drinking influenced Chinese college students’ binge drinking. On the basis of the extant literature, we proposed that descriptive norms regarding peer drinking would be positively associated with higher levels of binge drinking among first-and second-year Chinese college students (H1).

### The mediating role of enhancement drinking motives

Researchers have focused on the role of motivational mechanisms in the association between descriptive norms regarding peer drinking and alcohol consumption, namely, drinking motives (Carrus et al., 2016). The motivational model of alcohol use (Cox and Klinger, 1988; Cooper, 1994) postulates four different drinking motives according to the source (i.e., internal or external) and valence (i.e., positive or negative) of anticipated reinforcement: social motives (external positive reinforcement; e.g., drinking to affiliate), enhancement motives (internal positive reinforcement; e.g., drinking to enhance positive emotions), conformity motives (external negative reinforcement; e.g., drinking to avoid social rejection), and coping motives (internal negative reinforcement; e.g., drinking to alleviate negative emotions). When these drinking motives are evaluated simultaneously, the enhancement motives are the most robust predictor of drinking (Stevenson et al., 2019) and heavy drinking (Kuntsche et al., 2005; Müller and Kuntsche, 2011; Lannoy et al., 2019). Considering that heavy drinking itself is considered a precursor of adult drinkers’ alcohol use problems (Dumas et al., 2013), enhancement motives may be an important motive to study and the target for intervention among college students.

The motivational model of alcohol use (Cox and Klinger, 1988; Cooper, 1994) depicts drinking motives as the most proximal determinants of alcohol consumption through which the influences of more distal factors are mediated. In line with this model, Carrus et al. (2016) found that the enhancement motives mediated the association between descriptive norms regarding peer drinking and alcohol intake among 13-to 22-year-old adolescents. Moreover, Müller and Kuntsche (2011) showed that the enhancement motives functioned as the strongest mediator in the effects of perceived parental drinking behaviors on adolescents’ own frequency of drunkenness. Taken together, these studies point to the critical role of enhancement motives—as a mediator—in the link between perceived others’ alcohol drinking and individuals’ own (heavy) drinking. Combined with the motivational model and the evidence that descriptive drinking norms are linked to alcohol consumption *via* enhancement motives, we hypothesized that descriptive norms regarding peer drinking would have a positive effect on enhancement motives, which would, in turn, increase binge drinking (H2).

### The moderating role of alcohol resistance self-efficacy

Alcohol resistance self-efficacy, the belief about one’s ability to resist pro-alcohol influences (Ellickson et al., 2003; Shih et al., 2012), may play a pivotal role in individuals’ alcohol use. Previous studies, mainly conducted in adolescents, have shown that higher levels of alcohol resistance self-efficacy were associated with lower odds of alcohol initiation (Shih et al., 2012), less alcohol intake (Choi et al., 2013; Shih et al., 2017), and less frequent heavy drinking (Tucker et al., 2008). It is noteworthy that alcohol resistance self-efficacy is not identical to drinking refusal self-efficacy (Jang et al., 2013) such that the former captures individuals’ confidence of resisting alcohol offers in the face of pro-alcohol influences (Ellickson et al., 2003; Carpenter and Howard, 2009). Considering that lowerclassmen may spend much time on adjusting to the campus life and indicate less mature competence of interpersonal skills (Shin et al., 2019), they might encounter various sources of pro-alcohol pressures (e.g., observations of peer drinking in social situations). As such, it is crucial to understand whether alcohol resistance self-efficacy could influence students’ responses in the context of elevated peer drinking norms during the critical developmental stage.

The theory of normative social behavior (TNSB; Rimal and Real, 2005; Chung and Rimal, 2016) extends the social norms theory (Berkowitz, 2005) by depicting the conditions under which descriptive norms influence individuals’ behaviors (e.g., binge drinking). According to the TNSB (Rimal and Real, 2005; Chung and Rimal, 2016), the effects of descriptive norms on behaviors should be understood in the context of meaningful moderators because people do not act solely on the basis of what others are doing in a given situation, they also behave defiantly and refuse to go along with the majority. The TNSB illustrates specific variables under individual (e.g., self-efficacy), behavioral (e.g., addictiveness of a behavior), and contextual (e.g., external monitoring) categories, which may affect the relationship between descriptive norms and behaviors (Chung and Rimal, 2016). In line with the TNSB, Jang et al. (2013) found an interaction effect of drinking refusal self-efficacy and descriptive norms regarding peer drinking on alcohol consumption in a sample of 14-to 17-year-old adolescents. Specifically, the positive effects of descriptive norms regarding peer drinking on alcohol consumption were strengthened among adolescents with low drinking refusal self-efficacy as compared to those with high drinking refusal self-efficacy. However, this study focused primarily on the moderating role of drinking refusal self-efficacy (rather than alcohol resistance self-efficacy) in the relation between descriptive peer drinking norms and non-heavy alcohol consumption among adolescents. It is uncertain how the perceived ability to resist pro-alcohol pressures influences the effects of descriptive peer drinking norms on excessive alcohol consumption among the lowerclassmen, who might experience changes from abstinence to drinking (Lindgren et al., 2018). The current study aimed to fill this gap by examining alcohol resistance self-efficacy as a moderator in the relation between descriptive peer drinking norms and binge drinking in a Chinese lowerclassmen sample. On the basis of the above theory and studies, we proposed that alcohol resistance self-efficacy would moderate the relationship between descriptive norms regarding peer drinking and college students’ own binge drinking. Specifically, students with low alcohol resistance self-efficacy whose binge drinking would be more likely to be influenced by descriptive peer drinking norms when compared to those with high alcohol resistance self-efficacy (H3).

### Current study

**Figure 1:**
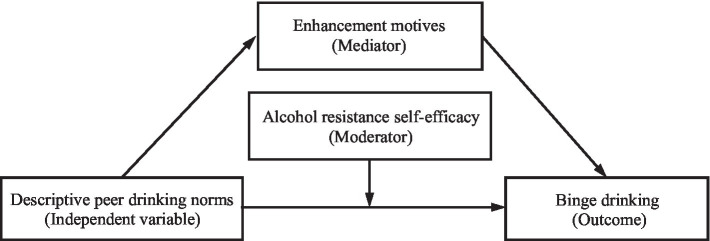
The hypothesized model.

The purpose of this study was to replicate and extend the results of previous studies (Jang et al., 2013; Carrus et al., 2016) concerning descriptive norms regarding peer drinking, enhancement motives, alcohol resistance self-efficacy, and alcohol consumption with two main distinctions. First, we examined the effects of descriptive norms regarding peer drinking on binge drinking behavior among first-and second-year college students who reported heavy drinking within the past year in Chinese culture in which excessive drinking is a widely accepted behavior and a salient norm among college students (Newman et al., 2014; Sun et al., 2018). Second, we simultaneously incorporated enhancement motives as a mediator and alcohol resistance self-efficacy as a moderator in the association that descriptive peer drinking norms have with binge drinking. Study findings would facilitate a better understanding of the mechanisms that explain how descriptive norms regarding peer drinking influence binge drinking behavior, and might help improve the efficacy of programs that prevent problematic drinking for the lowerclassmen in cultures like China. The effects of sex and age were controlled in our study because alcohol consumption has been shown to differ in youths depending on both sex and age. Regarding sex differences, male college students drink more on average and engage in heavy episodic drinking more often than female college students (Neighbors et al., 2008; Leigh and Neighbors, 2009; Pedersen, 2013). Regarding age differences, the trends for current drinking and heavy episodic drinking increase with age in the late teens (15–19 years old) and early twenties (20–24 years old), and decrease thereafter (World Health Organization, 2018). Based on previous research, we proposed the following hypotheses:

*H1*: Stronger descriptive norms regarding peer drinking would be associated with higher levels of binge drinking (see Figure 1 for a conceptual model).

*H2*: The stronger descriptive norms regarding peer drinking would predict a greater tendency to drink for enhancement motives, which would in turn predict higher levels of binge drinking.

*H3*: Alcohol resistance self-efficacy would moderate the effects of descriptive peer drinking norms on binge drinking. Specifically, the positive association between descriptive norms and binge drinking would be stronger for students who reported lower levels of alcohol resistance self-efficacy.

## Materials and methods

### Participants and procedure

Five hundred and nineteen first-and second-year full-time college students (*M*_age_ = 19.19, *SD* = 0.98, 57.8% freshmen, 49.3% female) from four universities in Zhejiang province, China, participated in the current study. The participants were recruited *via* posters, flyers, and personal communications. The participation was voluntary, and no incentives were provided. Before data collection, participants were asked if they had engaged in heavy episodic drinking during the past 12 months. The heavy episodic drinking was defined as “consuming 4 or more alcoholic drinks for females, and 5 or more for males within a two-hour period” (National Institute on Alcohol Abuse and Alcoholism, 2015). The participants were displayed with standard drink images and texts that defined one drink as a can (250 ml) of beer, a glass (100 ml) of grape wine or Chinese rice wine, or a small cup (20 ml) of 80-proof liquor (Ji, 2010). Only the students who reported at least one time of heavy episodic drinking were included. Students who reported that they did not engage in heavy episodic drinking (e.g., those who had a sip of alcohol) within the past year were excluded from the study. All the participants were native Chinese speakers.

In quiet classroom settings, a paper-and-pen survey was administered to the participants, and the anonymous and confidential nature of the data to be collected was emphasized. Before filling out the questionnaires, participants were informed of the scientific purpose of this study and their right to withdraw at any time. The participants were asked not to leave any item blank and to check their responses after completion of the questionnaires. No questions were left blank by the participants. This study was approved by the ethics board of the Department of Psychology at Zhejiang Normal University, China. The participants provided their written informed consent prior to data collection.

### Measures

#### Descriptive norms regarding peer drinking

Items from the Drinking Norms Rating Form (DiBello et al., 2018) were employed to assess descriptive norms regarding peer drinking. The items included: (a) “How many of your close friends drink alcohol?” (b) “How many of your friends get drunk on a regular basis (at least once a month)?” and (c) “How many of your close friends drink primarily to get drunk?” Each item was rated on a 5-point scale ranging from 1 (*none*) to 5 (*nearly all*). The items were averaged to form a composite score of descriptive norms regarding peer drinking. Cronbach’s alpha of the measure was 0.63 in the current sample.

#### Enhancement motives

The enhancement subscale of the Chinese version of Drinking Motives Questionnaire-Revised (DMQ-R; Cooper, 1994; Cheng et al., 2016) was used to assess enhancement motives for drinking. The enhancement subscale consisted of five items (e.g., to get high). Participants were instructed to think of all the times they had drunk alcohol in the past 12 months, and to rate their frequency of drinking for each reason. Responses were given on the 5-point scale ranging from 1 (*Almost Never/Never*) to 5 (*Almost Always/Always*). Items were averaged to create a single-item index of enhancement motives. Cronbach’s alpha of the subscale was 0.93 in this sample.

#### Alcohol resistance self-efficacy

The alcohol resistance self-efficacy was assessed using a measure from the Project ALERT study (Ellickson et al., 2003). Participants were asked three questions: “Suppose you are offered alcohol and you do not want to use it. What would you do in these situations: (a) your best friend is drinking alcohol; (b) you are bored at a party; and (c) all your friends at a party are drinking alcohol?” The items included a conditional statement specifying that the respondent did not want to use alcohol. This phrasing could reduce the likelihood of potentially confounding students’ confidence of resisting alcohol offers with their notions of desire or willingness to use alcohol (Carpenter and Howard, 2009). Each item was rated on a 4-point scale ranging from 1 (*I would definitely drink*) to 4 (*I would definitely not drink*). Items were averaged with higher scores indicating that students were more confident in their ability to resist alcohol offers. Cronbach’s alpha of the measure was 0.74 in the present sample.

#### Frequency of binge drinking

Consistent with the definition of binge drinking provided by National Institute on Alcohol Abuse and Alcoholism (2015), the binge drinking question was phrased: “During the last 12 months, how often did you have 5 or more (males) or 4 or more (females) drinks containing any kind of alcohol within a 2-h period?” Responses were given on a 10-point scale ranging from 0 (*none*) to 9 (*everyday*). The higher scores represented more frequent binge drinking during the past 12 months.

### Statistical analyses

Data analyses were conducted *via* SPSS 22.0. We firstly checked normality of the study variables. The distribution of binge drinking scores was positively skewed. A Log 10 transformation was used, and the values of skewness and kurtosis were in acceptable ranges (skewness = 1.92, kurtosis = 3.86; Weston and Gore, 2006). To assess potential multicollinearity among the predictors, the variance inflation factors (VIF) were estimated. Our analysis indicated that the VIF indices for predictors were 1.37 or below, indicating no serious multicollinearity (Li et al., 2022).

The zero-order correlations were computed to investigate the relationships among descriptive norms regarding peer drinking, enhancement motives, alcohol resistance self-efficacy, and binge drinking. Hayes’ PROCESS macro (version 3.5) for SPSS (Hayes, 2018) was used to examine whether the association between descriptive norms regarding peer drinking and frequency of binge drinking was mediated by enhancement motives and was moderated by alcohol resistance self-efficacy. The descriptive norms regarding peer drinking were entered as an independent variable (X), enhancement motives as a mediator (M), alcohol resistance self-efficacy as a moderator (W), and binge drinking frequency as the dependent variable (Y). The estimates of 95% confidence intervals (*CI*s) of standardized effects were calculated using 1,000 bootstrapped samples. A 95% *CI* that did not contain zero provided evidence of a significant effect (Preacher and Hayes, 2008). Age and sex were included as model covariates. Considering that a significant main effect was found for university variable on levels of descriptive norms regarding peer drinking [*F*_(3, 515)_ = 4.28, *p* < 0.01], the university variable was dummy coded and was added as covariates.

## Results

### Preliminary analyses

The means, standard deviations, and bivariate correlations for the variables are presented in Table 1. Here, the descriptive norms regarding peer drinking were positively associated with both enhancement motives and frequency of binge drinking. The enhancement motives were positively associated with frequency of binge drinking. Moreover, the alcohol resistance self-efficacy was negatively associated with descriptive norms regarding peer drinking, enhancement motives, and frequency of binge drinking.

**Table 1:** Means (*M*), standard deviations (*SD*), and correlations among variables.

	*M* ± *SD*	1	2	3
1. Descriptive peer drinking norms	2.18 ± 0.64	–		
2. Enhancement motives	2.23 ± 1.11	0.21^***^	–	
3. Alcohol resistance self-efficacy	2.09 ± 0.60	−0.12^**^	−0.29^***^	–
4. Frequency of binge drinking	2.61 ± 1.17	0.32^***^	0.27^***^	−0.19^***^

*N* = 519. ^**^p < 0.01; ^***^p < 0.001.

### Testing for the hypothesized model

To examine the hypothesized model (see Figure 1), Hayes’ PROCESS macro Model 5 (Hayes, 2018) was performed. The results revealed that descriptive norms regarding peer drinking had positive effects on enhancement motives (*β* = 0.20, *p* < 0.001, see Model 1 of Table 2), which, in turn, positively predicted the frequency of binge drinking (*β* = 0.19, *p* < 0.001, see Model 2 of Table 2). Moreover, the direct effects of descriptive norms regarding peer drinking on frequency of binge drinking were significant (*β* = 0.23, *p* < 0.001, see Model 2 of Table 2). Bootstrapping analyses showed that the indirect effects of descriptive norms regarding peer drinking on frequency of binge drinking *via* enhancement motives were significant (indirect effect = 0.04, *SE* = 0.01, 95%*CI* = [0.02, 0.07]), accounting for 14.81% of the total effect (0.27). In sum, enhancement motives partially mediated the effects of descriptive norms regarding peer drinking on frequency of binge drinking.

**Table 2:** Testing the mediating effect of enhancement motives and the moderating effect of alcohol resistance self-efficacy in the relation between descriptive peer drinking norms and binge drinking frequency.

Predictor	Model 1: Enhancement motives				Model 2: Binge drinking frequency			
	*β*	*SE*	*t*	95%*CI*	*β*	*SE*	*t*	95%*CI*
Sex	−0.05	0.09	−0.56	[−0.23, 0.13]	0.41	0.08	5.15^***^	[0.25, 0.57]
Age	−0.03	0.05	−0.66	[−0.12, 0.06]	0.01	0.04	0.23	[−0.07, 0.09]
University 2	0.12	0.13	0.93	[−0.13, 0.37]	0.14	0.12	1.25	[−0.08, 0.37]
University 3	0.15	0.12	1.28	[−0.08, 0.39]	0.16	0.11	1.45	[−0.06, 0.38]
University 4	0.17	0.10	1.65	[−0.03, 0.37]	0.23	0.12	1.94	[−0.002, 0.47]
DPDN	0.20	0.04	4.65^***^	[0.12, 0.29]	0.23	0.04	5.58^***^	[0.15, 0.31]
EM					0.19	0.04	4.62^***^	[0.11, 0.28]
ARSE					−0.08	0.04	−1.82	[−0.16, 0.01]
DPDN × DRSE					−0.09	0.04	−2.45^*^	[−0.17, −0.02]
*R* ^2^	0.05				0.21			
*F*	4.65^***^				15.29^***^			

*N* = 519. The beta coefficients are standardized coefficients except for sex and university variable. Each column is a regression model that predicts the outcome variable at the top of the column. DPDN, Descriptive peer drinking norms; EM, Enhancement motives; ARSE, Alcohol resistance self-efficacy. Sex was dummy coded such that 0 = female and 1 = male. The university variable was dummy coded such that students from the university (University 1) with the lowest level of descriptive peer drinking norms were the reference group. ^*^p < 0.05; ^***^p < 0.001.

**Figure 2:**
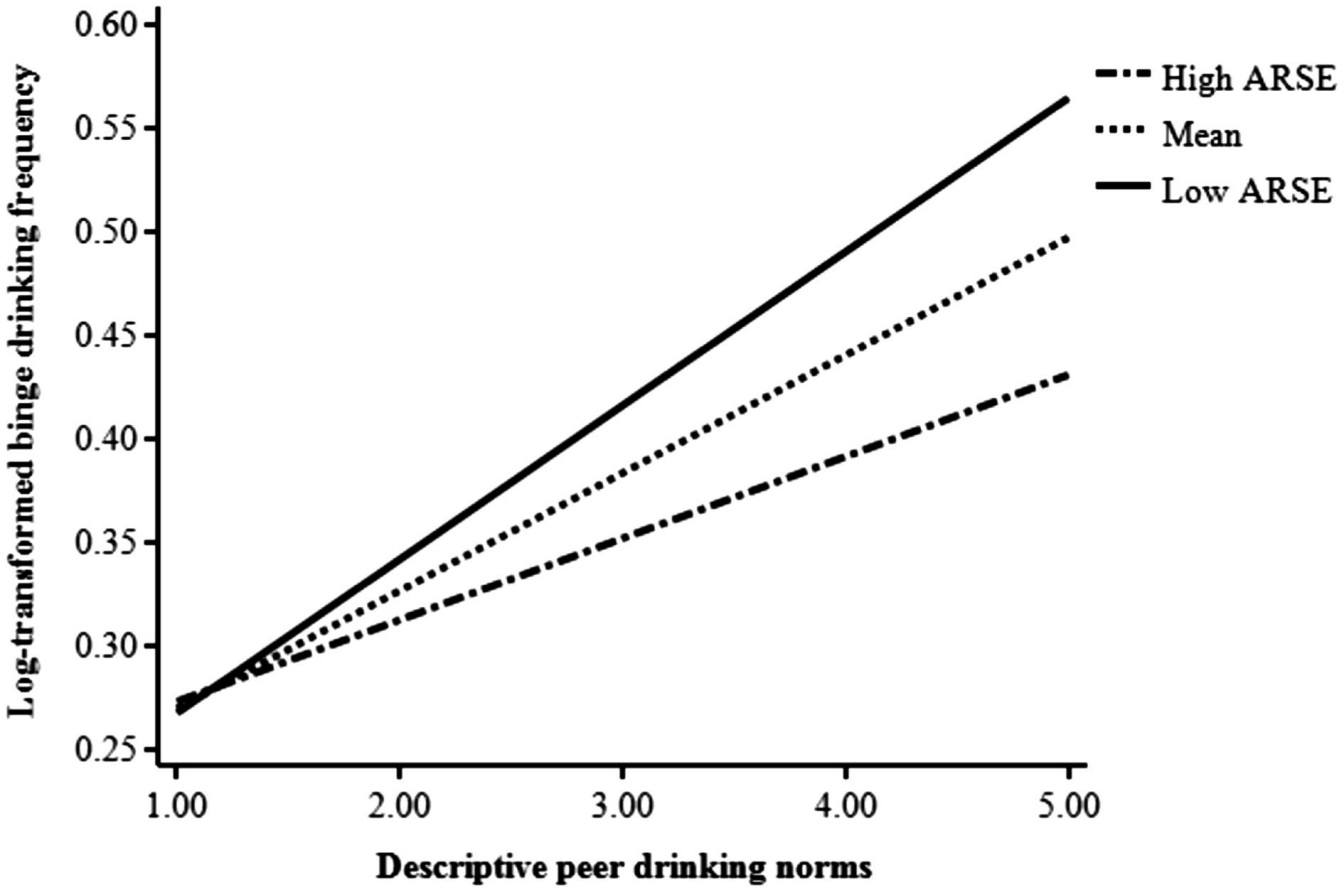
Alcohol resistance self-efficacy (ARSE) moderated the effects of descriptive peer drinking norms on binge drinking frequency.

In addition, alcohol resistance self-efficacy was shown to significantly moderate the association between descriptive norms regarding peer drinking and binge drinking frequency (*β* = −0.09, *p* = 0.02, see the Model 2 of Table 2). The relation between descriptive norms regarding peer drinking and binge drinking frequency was graphed in participants at low and high alcohol resistance self-efficacy (1 *SD* below and above the mean, respectively; see Figure 2). Among individuals with high alcohol resistance self-efficacy, the descriptive norms regarding peer drinking were significantly positively associated with binge drinking frequency (*β*_simple_ = 0.14, *t* = 2.37, *p* = 0.02). However, the strength of such positive association was stronger for those reporting low alcohol resistance self-efficacy (*β*_simple_ = 0.32, *t* = 6.02, *p* < 0.001).

## Discussion

The current study aimed to examine the association between descriptive peer drinking norms and binge drinking, and to probe the underlying mechanisms in this association. Specifically, we aimed to explore the mediating role of enhancement motives and the moderating role of alcohol resistance self-efficacy in the relationship between descriptive peer drinking norms and binge drinking with a sample of first-and second-year Chinese college students who had engaged in heavy episodic drinking within the past year.

### Associations of descriptive norms regarding peer drinking with alcohol consumption

This study replicates and extends previous work with college students in Western countries (Park et al., 2009; Franca et al., 2010), such that first-and second-year Chinese college students who had a history of binge drinking and reported higher levels of descriptive norms regarding peer drinking tended to report more frequent binge drinking during the last 12 months. This finding supports our first hypothesis and the social norms theory that individuals’ behaviors are influenced by their perceptions of behaviors and attitudes of reference groups (Berkowitz, 2005). In China, frequent toasting during meals with friends is a deep-rooted cultural practice, and the cultural norms encourage mutual intoxication during meals to build and strengthen social relationships among adults, including college students (Cheng et al., 2017; Sun et al., 2018). Thus, heavy episodic drinking may be perceived as a normative behavior of reference groups (e.g., peer groups) among Chinese college students. Additionally, compared with upperclassmen (i.e., junior and senior), the lowerclassmen (i.e., freshman and sophomore) who are in the transition from high school to college may spend more time on adjusting to the campus life and reveal less mature competence of interpersonal skills (Shin et al., 2019). Taken together, for the first-and second-year college students, the perceptions or observations of peers’ alcohol use may become an important way to obtain information about the normative way to behave in a university campus, which might promote them to engage in heavy alcohol use.

### The mediating role of enhancement motives

As hypothesized and consistent with previous studies (Müller and Kuntsche, 2011; Lannoy et al., 2019), this study revealed that stronger enhancement motives were associated with more frequent binge drinking. Also, this finding is in line with behavioral economic perspectives on college student drinking which have suggested that the lack of non-substance-related reinforcement may be an important risk factor for excessive alcohol consumption (Murphy et al., 2006) and that the more pleasurable feelings students obtain from non-substance-related activities, the less likely they engage in excessive alcohol use (Murphy et al., 2007). More importantly, the current study showed that drinking for enhancement motives was a significant mediator in the relation between descriptive norms regarding peer drinking and frequency of binge drinking among the first-and second-year college students. This finding supports our second hypothesis and provides evidence that drinking motives may be critical proximal factors of excessive alcohol consumption, and more distal factors, including peer drinking behaviors, indirectly influence binge drinking *via* drinking motives (Cox and Klinger, 1988; Kuntsche et al., 2005). Such finding is also consistent with previous studies that enhancement motives played a mediating role in the effects of descriptive drinking norms of peers and parents on alcohol consumption among adolescents (Müller and Kuntsche, 2011; Carrus et al., 2016). To sum up, the present findings suggest that first-and second-year college students with higher levels of descriptive peer drinking norms may appear to be more likely to drink in order to increase their positive emotions, which ultimately, in turn, motivates them to engage in binge drinking.

### The moderating role of alcohol resistance self-efficacy

Our study advances the extant literature by examining whether the association of descriptive peer drinking norms with binge drinking differs according to alcohol resistance self-efficacy, which includes a qualifying statement that may reduce potentially confounding confidence of resisting alcohol offers with notions of desire or willingness to use alcohol (Carpenter and Howard, 2009). After controlling for demographics and enhancement motives, descriptive peer drinking norms interacted with alcohol resistance self-efficacy to predict binge drinking frequency among first-and second-year Chinese college students. Specifically, although stronger descriptive peer drinking norms predicted a greater frequency of binge drinking among lowerclassmen with high alcohol resistance self-efficacy, this positive association was strengthened among those with low self-efficacy in resisting pro-drinking influences. Overall, these results support our third hypothesis, and are consistent with the findings of Jang et al. (2013) who found that the relation between descriptive norms and alcohol consumption was stronger among teenagers with low drinking refusal self-efficacy as compared to those with high drinking refusal self-efficacy. Our results are consistent with the theory of normative social behavior (TNSB; Rimal and Real, 2005; Chung and Rimal, 2016), which posits that the effects of descriptive norms on individuals’ behaviors vary across certain individual (e.g., self-efficacy), behavioral, or contextual factors. In sum, particularly in the high-risk context of pervasive peer drinking, lacking confidence or skills to resist pro-drinking influences might further heighten risks for frequent binge drinking among first-and second-year Chinese college student drinkers. In contrast, increased alcohol resistance self-efficacy might serve as a protective function against binge drinking for Chinese college students who perceived high prevalence of peer drinking.

### Practical implications

In line with findings from U.S. and European college students (McAlaney and McMahon, 2007; Franca et al., 2010; Cox et al., 2019), Chinese college students also tended to overestimate peers’ alcohol use (Sun et al., 2018). In light of our findings that descriptive norms regarding peer drinking may exert a positive effect on binge drinking, one approach to addressing the association between descriptive norms and college students’ problematic drinking could focus on changing the misperceptions of peer drinking behaviors. The widely used strategies are the norms-based drinking interventions, which posit that highlighting discrepancies in perceived and actual peer drinking and correcting normative perceptions could reduce individuals’ alcohol use (Dotson et al., 2015). Specifically, university administrators could facilitate social norms campaigns to correct Chinese college students’ misperceptions of the prevalence of students’ alcohol use that actually occurs in their campuses. Meanwhile, college mental health counselors could give personalized normative feedback (e.g., perceived drinking behaviors and actual drinking behaviors of students’ peers) when working with Chinese college students struggling with problematic drinking (Saxton et al., 2021).

Previous intervention research has developed some promising interventions of drinking motives, which concentrated on the illusory enhancement functions arising from heavy alcohol use and helped student drinkers identify their mood states and develop effective strategies of increasing pleasant emotions (Canale et al., 2015; Wurdak et al., 2016). Additionally, previous studies have implemented resistance self-efficacy enhancing activities as part of intervention programming for students who used alcohol or other drugs (Ellickson et al., 2003; Heyne and Bogner, 2009; Karatay and Baş, 2017). The activities to bolster students’ competence of resisting alcohol offers during drinking occasions include the use of phrases to say “no” to alcohol offers, and practice of desired alcohol resistance skills (Ellickson et al., 2003; Karatay and Baş, 2017). Our results suggest that such strategies tailored to enhancement motives and alcohol resistance self-efficacy may be effective to prevent the influence of descriptive norms regarding peer drinking on alcohol use, and may provide complementary ways to improve the efficacy of interventions targeted at descriptive norms.

### Limitations and future directions

The current study should be considered in the context of its limitations. First, this study utilized a cross-sectional rather than a longitudinal design. Despite the theoretical basis for conceptualizing the relations in the manner discussed above, it is clearly possible that increases in heavy drinking contribute to the elevated strength of descriptive peer drinking norms. Longitudinal studies are needed to better examine the influence of descriptive peer drinking norms on binge drinking among first-and second-year college students in future studies. Second, the findings from the small-size college student drinkers of one province may not be fully generalized to college students from other provinces of China or non-college samples of young adult drinkers, who may have distinctive drinking patterns and peer drinking norms due to their different social networks. In addition, the study variables (e.g., descriptive norms regarding peer drinking, enhancement motives, alcohol resistance self-efficacy, and binge drinking) were measured at individual-level among college students from four universities. The lack of information regarding university-level variables (e.g., sex composition, denomination) prevented an examination of the effects of university-level characteristics through cluster-based approaches (e.g., multilevel modeling; Gaete and Araya, 2017). Future studies should use larger and more diverse college student samples, and incorporate university-level variables to replicate the present findings. Finally, participants were instructed to report their levels of enhancement motives and frequency of binge drinking during the past 12 months instead of a shorter reference period. Longer reference periods may be linked to higher recall bias. Although using reference periods shorter than 12 months may induce other bias (e.g., underestimation of infrequent heavy drinkers and overestimation of abstainers; Gmel and Rehm, 2004), future studies are warranted to validate the current findings using shorter reference periods.

## Conclusion

In summary, the current study replicates and extends previous work on the relationship between descriptive peer drinking norms and binge drinking, and provides further evidence for mechanisms underlying this association among first-and second-year Chinese college students. These findings point to the potential value of alcohol intervention approaches including efforts to change enhancement drinking motives and increase ability of resisting alcohol offers from peers in the context of pervasive peer drinking among lowerclassmen during the critical transition into college.

## Data availability statement

The raw data supporting the conclusions of this article will be made available by the authors, without undue reservation.

## Ethics statement

The studies involving human participants were reviewed and approved by the ethics board of Department of Psychology at Zhejiang Normal University, China. The patients/participants provided their written informed consent to participate in this study.

## Author contributions

JC and YL designed the study, collected the data, drafted and revised the manuscript. YZ, JF, and LJ analyzed the data and revised the manuscript. All authors contributed to the article and approved the submitted version.

## Funding

This study was funded by the Philosophy and Social Science Planning Project of Zhejiang Province, China (No. 20NDQN266YB).

## Conflict of interest

The authors declare that the research was conducted in the absence of any commercial or financial relationships that could be construed as a potential conflict of interest.

## Publisher’s note

All claims expressed in this article are solely those of the authors and do not necessarily represent those of their affiliated organizations, or those of the publisher, the editors and the reviewers. Any product that may be evaluated in this article, or claim that may be made by its manufacturer, is not guaranteed or endorsed by the publisher.
